# Supplementary material for: The effect of body image dissatisfaction on goal-directed decision making in a population marked by negative appearance beliefs and disordered eating
Source: PLoS One. 2022 Nov 28;17(11):e0276750. doi: 10.1371/journal.pone.0276750 (PMC9704573; doi:10.1371/journal.pone.0276750)
Supplement: Attachment — Submitted filename: PLoS ONE - Author Response.pdf [file pone.0276750.s005.pdf]

Rebuttal letter - PONE-D-22-05219

The effect of body image dissatisfaction on goal-directed decision making in a population marked by negative appearance beliefs and disordered eating

Dear Editor,

We are grateful for the opportunity to enhance our manuscript based on your comments as well as those from the reviewer. Editor's and reviewer's remarks are in bold and our replies in normal typeface, with the manuscript paragraphs being in italics. For clarity in the following text (including mention of new paragraphs added to the manuscript), we use an author-date system for the references, but they will be (re)numbered in the actual manuscript. We also refer to line numbering from the previously submitted system-combined pdf document, which matches that of the reviewer, as well as line numbering in the new version of the manuscript in brackets.

Editor's remarks

1. **Please ensure that your manuscript meets PLOS ONE's style requirements, including those for file naming.**

   We were not able to achieve 300 DPI for Fig1 and S1 Fig due to the inherently limited DPI of the images used to construct the figures. All the other figures meet the 300 DPI criterion. Furthermore, we have used Arial font style within the figures and we converted all text to outlines in the .eps files where possible. We didn't change the font size to be between 8-12 as we deemed this to be too small to be readable, and arbitrary as the font size will be relative to the figure scaling used in the final print version of the article. We also removed figure (sub-)titles from the figures themselves, where it didn't impede figure interpretation, i.e Fig 4 and S2 Fig retained sub-titles to aid readability of the condition the figure refers to. Lastly, we have removed figures from the main body, keeping only the figure captions at the end of the paragraph where they are referenced.

   We have followed formatting guidelines for the title page (https://journals.plos.org/plosone/s/file?id=ba62/PLOSOne_formatting_sample_title_authors_affiliations.pdf) as well as the main body (https://journals.plos.org/plosone/s/file?id=wjVg/PLOSOne_formatting_sample_main_body.pdf).

2. **Please provide additional details regarding participant consent. In the ethics statement in the Methods and online submission information, please ensure that you have specified what type you obtained (for instance, written or verbal, and if verbal, how it was documented and witnessed). If your study included minors, state whether you obtained consent from parents or guardians. If the**

need for consent was waived by the ethics committee, please include this information.

At the end of line 192 (262), we added the following information regarding participant consent:

> *After being presented with a participant information sheet, participants gave explicit consent to take part in the study using an online consent form hosted on Qualtrics.*

3. **Thank you for stating the following financial disclosure: "Participants payment was funded by the Institute for Adaptive and Neural Computation, Informatics, University of Edinburgh.The funders had no role in study design, data collection and analysis, decision to publish, or preparation of the manuscript." At this time, please address the following queries:**
    a. **Please clarify the sources of funding (financial or material support) for your study. List the grants or organizations that supported your study, including funding received from your institution.**

       The funding for the online participants participation was provided by the Institute for Adaptive and Neural Computation, School of Informatics, University of Edinburgh and was not covered by a specific grant.
    b. **State what role the funders took in the study. If the funders had no role in your study, please state: "The funders had no role in study design, data collection and analysis, decision to publish, or preparation of the manuscript."**

       The funders had no role in study design, data collection and analysis, decision to publish, or preparation of the manuscript.
    c. **If any authors received a salary from any of your funders, please state which authors and which funders.**

       N/A
    d. **If you did not receive any funding for this study, please state: "The authors received no specific funding for this work."**

       The authors received no salary or other specific funding for this work.

   Please include your amended statements within your cover letter; we will change the online submission form on your behalf.
4. **Please include captions for your Supporting Information files at the end of your manuscript, and update any in-text citations to match accordingly.**

   We have added supporting information captions at the end of the manuscript. We have separated S2, S7 Tables and S1 Fig from the original supporting information document, while keeping the remaining text, figures and tables in S1 File.
5. **Please review your reference list to ensure that it is complete and correct. If you have cited papers that have been retracted, please include the rationale for doing so in the manuscript text, or remove these references and replace them with relevant current references. Any changes to the reference list should be mentioned in the rebuttal letter that accompanies your revised manuscript. If**

you need to cite a retracted article, indicate the article's retracted status in the References list and also include a citation and full reference for the retraction notice.

We corrected the date of publication in Foerde et al. reference to be 2021:

- Foerde K, Daw ND, Rufin T, Walsh BT, Shohamy D, Steinglass JE. Deficient Goal-Directed Control in a Population Characterized by Extreme Goal Pursuit. Journal of Cognitive Neuroscience. 2021;33(3):463–81

We also added the following references, as indicated in the changed paragraphs in response to reviewer comments:

- van Swieten MMH, Bogacz R, Manohar SG. Hunger improves reinforcement-driven but not planned action. Cogn Affect Behav Neurosci. 2021;21(6):1196–206
- Everitt BJ, Robbins TW. Drug Addiction: Updating Actions to Habits to Compulsions Ten Years On. Annu Rev Psychol. 2016;67(1):23–50.
- C. Barbarich-Marsteller N, W. Foltin R, Timothy Walsh B. Does Anorexia Nervosa Resemble an Addiction? CDAR. 2011;4(3):197–200.
- Otto AR, Gershman SJ, Markman AB, Daw ND. The Curse of Planning: Dissecting Multiple Reinforcement-Learning Systems by Taxing the Central Executive. Psychol Sci. 2013;24(5):751–61.
- Waldron EM, Ashby FG. The effects of concurrent task interference on category learning: Evidence for multiple category learning systems. Psychonomic Bulletin & Review. 2001;8(1):168–76.
- Tolman EC. Cognitive maps in rats and men. Psychological Review. 1948;55(4):189–208.
- O'Keefe J, Nadel L. The hippocampus as a cognitive map. Oxford : New York: Clarendon Press ; Oxford University Press; 1978.
- Gershman SJ, Markman AB, Otto AR. Retrospective revaluation in sequential decision making: A tale of two systems. Journal of Experimental Psychology: General. 2014;143(1):182–94.

We have also fixed any deviations from the Vancouver referencing style we could spot.

Reviewer

1. **The article would benefit from a clearer exposition of how their experiment and/or results differ from those which have previously used the 2-stage task in eating disorders (refs 27 and 30). Having read these papers, it is clear how your work fits into the literature, but it would benefit the reader if you could bring this out more in the manuscript. You may also want to mention papers looking at hunger and motivation in the 2-stage task given the overlap with EDs (e.g.https://link.springer.com/article/10.3758/s13415-021-00921-w).**

   We thank the reviewer for these suggestions. We now cite the work of Van Swieten et al. at the end of line 138 (190):

   > *Another study of potential interest is that on the effect of hunger on the two systems by Van Swieten et al. (2021). The authors provide evidence that*

hunger improves healthy participants' overall performance in the two-step decision task by increasing reliance on model-free control, without affecting model-based learning.

We don't discuss our results in light of Van Swieten et al. further, as we cannot establish the level of hunger in our groups.

We further expound on the methods and results differences between our study and those already there in the field. Upon reviewer's suggestions, we made the following changes:

in line 554-555 (701-705), we replaced:

This is also contradictory with previous studies that find no difference in model-free learning between groups in the monetary two-step task (27,30).

by:

This is also contradictory with previous studies that find no difference in model-free learning between AN and HC groups in the monetary two-step task (Foerde et al. 2021,Gillan et al. 2016) or increased reliance on model-free learning in a BED group (Voon et al. 2015).

We also added, at the end of line 562 (712):

, while the difference with Voon et al. 2015 study may also stem from a different parameterisation of the two systems. Unlike in Voon et al. (2015), our computational model does not include the parameter w that forces the two systems to trade-off against each other. In their case, the reported reduction in model-based learning necessarily results in an increase of model-free learning, while this is not necessarily the case for us.

In 123-124 (173-175), we changed:

It has been shown that healthy participants employ both model-free and model-based learning that trade off against each other (10).

to:

The two systems can be captured using a well-established two-step decision-making task based on a reinforcement learning paradigm, which shows that healthy participants employ both model-free and model-based learning that trade off against each other (Daw et al., 2011).

In 149-150 (207-209), we changed:

We employ two task conditions, one of which attempts to target the dissatisfaction with body image and its manifestation in decision making.

to:

We employ two task conditions: first, a standard monetary version (Daw et al., 2011), with a changed narrative to achieve higher engagement; second, a body-image related version, which aims to target dissatisfaction with body image and its manifestation in decision making.

In line 153-154 (212-215), we changed:

Secondly, the condition mirrors the food-related task of Foerde et al. (27), aiming to support the domain-general nature of the goal-directed deficit.

to:

Secondly, our design intends to test whether the goal-directed deficit may extend beyond the domain of purely monetary tasks, into that more specific to negative appearance beliefs. This partly mirrors Foerde et al. (2021), but unlike the authors' objective ours may not necessarily capture whether the said deficits are domain-general.

In line 239-240 (313-314), we changed:

Selected participants were asked to complete a two-step decision-making task in order to quantify model-based and model-free learning contribution (10).

to:

Selected participants were asked to complete an established two-step decision-making task shown to quantify model-based and model-free learning contribution (Daw et al., 2011).

In line 276-281 (363-369), we changed:

The neutral (NT) condition is exactly like in the Fig 1, with a pirate coin as a reward (Fig 3A). The other condition, the body image dissatisfaction (BID) condition, differs from the neutral in that the reward is a pirate coin next to a body type that the participant selected as the most similar to their own (Fig 3C). The aim of the BID condition is to gauge the effects of body image dissatisfaction on decision-making.

to:

The neutral (NT) condition is exactly like in Fig 1, with a pirate coin as a reward (Fig 3A), and, aside from the narrative change, follows exactly from Daw et al (2011) and Gillan et al. (2016). The other condition, the body image dissatisfaction (BID) condition, aims to gauge the effects of body image dissatisfaction on decision-making. The BID condition is different from the neutral condition in that the reward is a pirate coin next to a body type that the participant selected as most similar to their own (Fig 3C).

In line 549-550 (692-697), we changed:

These results confirm the set hypotheses regarding model-based learning.

to:

These results support the set hypotheses regarding model-based learning, suggesting that goal-directed deficits extend beyond purely monetary tasks and are more pronounced in the domain of body image preoccupation. This mirrors previously established results of further goal-direct deficits in AN when the task is food-related (Foerde et al., 2021), which demonstrate that such deficits affect multiple domains of decision making.

2. **In the discussion (around line 630) you argue that the BID condition could not be impairing learning because model-free learning was unaffected. Have you considered the possibility that the body representation was specifically distracting to ED participants (who are more preoccupied with body images), resulting in mind wandering, rumination etc? If so, given that model-based learning is known to be sensitive to cognitive load, whereas model-free**

learning is relatively insensitive (see https://journals.sagepub.com/doi/10.1177/0956797612463080?url_ver=Z39.88-2003&rfr_id=ori:rid:crossref.org&rfr_dat=cr_pub%20%200pubmed), is it possible that this could account for the findings you report? In other words, rather than triggering habitual behaviour (as described around line 586) could the BID condition have instead hampered model-based learning? If so this should be acknowledged in the manuscript.

We recognise this point and agree with the reviewer that our results could be accounted for by model-based being sensitive to cognitive load.

Following this, In line 583-585 (743-746), we changed:

> *The fear of gaining weight and associated with it goal of either losing or not gaining weight set as a goal, evolves over time into a rigid and habitual body checking.*

To:

> *The fear of gaining weight and a goal of either losing or not gaining weight, evolves over time into a rigid and habitual body checking, which may be based in a shift away from the model-based system, in a similar fashion as in addiction (Everitt et al. 2016) where, overtime, such goal becomes overvalued (Barbarich-Marsteller et al. 2011).*

Then in line 630-632 (797-804), we changed:

> *From this, we would expect reward valuation within the model-based strategy to be similarly unaffected, since we do not see a reason why the negative effect silhouettes would be selective to one type of reward learning.*

To:

> *However, we recognise that the BID condition may have been distracting to the ED participants, due to body image preoccupation, and lead them to ruminate about their body, or to allocate more mental resources to the displayed silhouettes. As such, the BID condition could be interpreted as a modulator of the availability of cognitive resources, which has been shown to control the extent to which healthy participants employ model-based strategy (Otto et al. 2013). As shown by Otto et al. (2013), when performance of the two-step task is systematically disrupted with a high working-memory load component (a numerical stroop task (Waldron and Ashby 2001)), participants reduce their reliance on the model-based system.*

In line 593 (758), we added right indentation.

In line 632 (804), we changed '*inversive*' to *'inverse'*.

3A. Line 110 – consider referencing Tolman 1948 (https://pubmed.ncbi.nlm.nih.gov/18870876/), where the idea of model-based learning originated.

In line 110-112 (142-146), change:

It is usually characterised by forming an internal model of the environment that describes which states and actions will bring about the best results over prolonged time.

To:

It is usually characterised by forming an internal model of the environment that describes which states and actions will bring about the best results over prolonged time (Dolan and Dayan, 2013), an idea originally put forward by Tolman (1948) who considered animals forming a 'field map of the environment" while in a maze, later reframed as a 'cognitive map' (O' Keefe and Nadel, 1978).

3B. Line 113/114 'without much deliberation' this phrase is confusing. It is unclear if deliberation refers to conscious or unconscious processes. It is not clear to me that there is any deliberation in model-free decision making as the action with the highest value is simply selected from representations in the striatum.

We agree with the reviewer and have removed 'without much deliberation', resulting in a change in lines 112-114 (146-148), from:

On the other hand, habitual decision-making is associated with responding to stimuli in the environment in an automatic manner, without much deliberation, usually repeating those actions that immediately yield the best results.

To:

On the other hand, habitual decision-making is associated with responding to stimuli in the environment in an automatic manner, usually repeating those actions that immediately yield the best results.

3C. Line 115/7 – it has been demonstrated by several papers (e.g. https://content.apa.org/record/2012-32653-001) that model-free learning can still be influenced by model-based information. The manuscript should reflect this as model-free valuations can take into account the probabilistic nature of the environment, albeit without an internal model.

At the end of line 122 (155), we added:

To make the best of accuracy and computational efficiency, the two systems may in fact interact with each other, rather than compete for cognitive control, where the model-based system trains the model-free system by simulating the environment. Such a simulation produces transitions and rewards from which the model-free system can then learn (Gershman et al. 2014).

3D. Line 120 – model-free learning is influenced by more than just the previous outcome ('last experience'). It may be influenced by hundreds of outcomes. Consider instead 'based on previous experience'. Similarly on line 122 – previous events.

We follow the suggestion and change lines 119-121 (152-154), from:

The habitual behaviour employs model-free learning, which updates a running score of possible states and actions, based on the last experience, without registering any hidden structures in the environment.

To:

The habitual behaviour employs model-free learning, which updates a running score of possible states and actions, based on previous experience, without registering any hidden structures in the environment.

3E. Consider adding a line about the implications of your work in the abstract.

We add the following at the end of the abstract:

This finding suggests general deficits in deliberate control in this population, leading to habitual, compulsive-like behaviours (body checking) dominating the experience. Importantly, the results may inform treatment approaches, which could focus on enhancing the reliance on goal-directed decision making to help cope with unwanted behaviours.

4. Throughout the manuscript there are several small typos and occasional sentences with clunky grammar. Some of the errors are listed below, but it would be worth proofing the entire manuscript again to correct any unfortunate niggles:

We have proofread the manuscript and any additional changes are reflected in the ‘tracked changes’ copy of the manuscript.

4A. Line 38 – removal of can – they do result in many deaths.

We removed ‘can’ from ‘*these problems can result*’.

4B. Line 86 – grammar – such links or such a link.

We changed ‘*such link*’ to ‘*such a link*’.

4C. Line 92 – 10 papers is not ‘a couple’ it is 10.

We changed ‘a *couple papers*’ to ‘*eleven papers*’

4D. Line 468 – formatting error – remove line break in mid-sentence.

We removed the line break in mid-sentence.

4E. Line 656 – on rather than o.

We changed ‘*o a clinical population*’ to ‘*on a clinical population*’.
